# Oriental Mouth Toilet

**Published:** 1901-04-15

**Authors:** 


ORIENTAL MOUTH TOILET.—The oral toilet of a Chinese mandarin is described. On rising he proceeds to take a large bowl, a silver tongue scraper and a tooth brush. Then ensues a long operation, consisting of rinsing and gargling, scraping the tongue and brushing the teeth, all done with minute care. We must not suppose that the teeth or their functions are thought lightly of in the Far East. A Japanese poet thus sings: "It is the function of the mouth to nourish the whole body. With what wisdom has it been fashioned! This noble gateway can open more or less wide, according to its need. Within we find the servants—the teeth, which crush and tear the carrots, turnips, radishes and all sorts of food, while the other servant—the tongue, tells whether the food is good or bad, and passes it on to the stomach. Its examination is so rigorous that not even a little stone in the rice can attempt to pass. When old age comes the teeth depart out of consideration for the enfeebled stomach, lest by their inconsiderately eating too much strong food they should over-tax its powers." This is a heretical theory for the dentist, although with such an indigestible diet as the foregoing it is perhaps necessary.—*Dental Record.*

